# [(*E*)-1-(Naph­thalen-2-yl)ethyl­idene](naph­thalen-1-ylmethyl)amine

**DOI:** 10.1107/S1600536812041414

**Published:** 2012-10-20

**Authors:** Sofia M. Bruno, José A. Fernandes, Isabel S. Gonçalves, Filipe A. Almeida Paz

**Affiliations:** a Department of Chemistry, University of Aveiro, CICECO, 3810-193, Aveiro, Portugal

## Abstract

The title compound, C_23_H_19_N, was obtained unexpectedly from the reaction of [Eu(nta)_3_(PzPy)] {Hnta = 1-(2-naphtho­yl)-3,3,3-trifluoro­acetone and PzPy = 2-[3(5)-pyrazol­yl]pyridine} with 1-naphthyl­methyl­amine. The 1- and 2-naphthyl groups are essentially planar [r.m.s. deviations of 0.007 and 0.011 Å, respectively] and subtend angles of 38.69 (11) and 16.50 (11)°, respectively, with the central CH_3_—C=N—CH_2_ unit, which is also almost planar [r.m.s. deviation = 0.002 Å]. In the crystal, the mol­ecules are disposed in zigzag-type fashion, forming layers perpendicular to [100]. Weak supra­molecular C—H⋯π inter­actions contribute to the packing forces.

## Related literature

For general background to aldimidines and ketimines, see: Norton *et al.* (1954 ▶); Hampe *et al.* (2004 ▶) and references cited therein; Kumar *et al.* (2008 ▶). For general background to β-diketonates, see: Bruno *et al.* (2008 ▶). Filyakova *et al.* (1996 ▶).
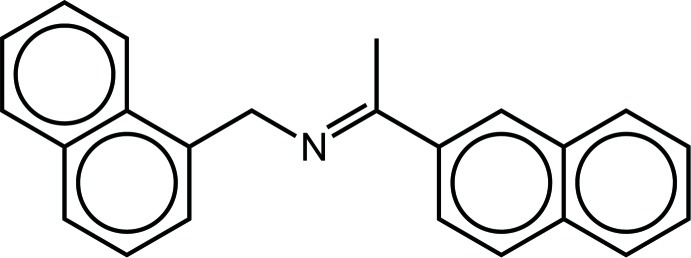


## Experimental

### 

#### Crystal data

C_23_H_19_N
*M* *_r_* = 309.39
Triclinic, 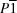

*a* = 6.6304 (5) Å
*b* = 7.7772 (5) Å
*c* = 16.7587 (9) Å
α = 77.655 (3)°
β = 87.969 (2)°
γ = 85.734 (3)°
*V* = 841.69 (9) Å^3^
*Z* = 2
Mo *K*α radiation
μ = 0.07 mm^−1^
*T* = 296 K
0.17 × 0.07 × 0.04 mm

#### Data collection

Bruker X8 Kappa APEXII CCD diffractometer
Absorption correction: multi-scan (*SADABS*; Sheldrick, 1998 ▶) *T*_min_ = 0.988, *T*_max_ = 0.997
14750 measured reflections
2986 independent reflections
2021 reflections with *I* > 2σ(*I*)
*R*_int_ = 0.045

#### Refinement

*R*[*F* ^2^ > 2σ(*F* ^2^)] = 0.044
*wR*(*F* ^2^) = 0.121
*S* = 1.02
2986 reflections
218 parameters
H-atom parameters constrained
Δρ_max_ = 0.13 e Å^−3^
Δρ_min_ = −0.16 e Å^−3^

### 

Data collection: *APEX2* (Bruker, 2006 ▶); cell refinement: *SAINT-Plus* (Bruker, 2005 ▶); data reduction: *SAINT-Plus*; program(s) used to solve structure: *SHELXTL* (Sheldrick, 2008 ▶); program(s) used to refine structure: *SHELXTL*; molecular graphics: *DIAMOND* (Brandenburg, 2009 ▶); software used to prepare material for publication: *SHELXTL*.

## Supplementary Material

Crystal structure: contains datablock(s) global, I. DOI: 10.1107/S1600536812041414/nr2032sup1.cif

Structure factors: contains datablock(s) I. DOI: 10.1107/S1600536812041414/nr2032Isup2.hkl

Supplementary material file. DOI: 10.1107/S1600536812041414/nr2032Isup3.cdx

Supplementary material file. DOI: 10.1107/S1600536812041414/nr2032Isup4.cml

Additional supplementary materials:  crystallographic information; 3D view; checkCIF report

## Figures and Tables

**Table 1:** Selected short distance interactions (Å, °) *Cg*1, *Cg*2 and *Cg*3 are the centroids of the C18–C23, C3–C8 and C6–C12 rings, respectively,

*D*—H⋯*A*	*D*—H	H⋯*A*	*D*⋯*A*	*D*—H⋯*A*
C9—H9⋯*Cg*1^i^	0.93	3.00	3.5739 (18)	122
C16—H16⋯*Cg*2^ii^	0.93	2.84	3.5147 (18)	130
C17—H17⋯*Cg*3^ii^	0.93	2.84	3.5626 (19)	135

Symmetry codes: (i) 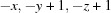; (ii) 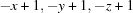.

**Fig. 1.**
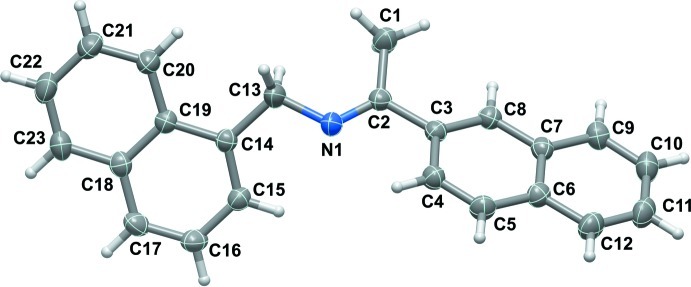
Asymmetric unit of the title compound showing all non-hydrogen atoms represented as thermal displacement ellipsoids drawn at the 30% probability level, and hydrogen atoms as small spheres with arbitrary radius.

**Fig. 2.**
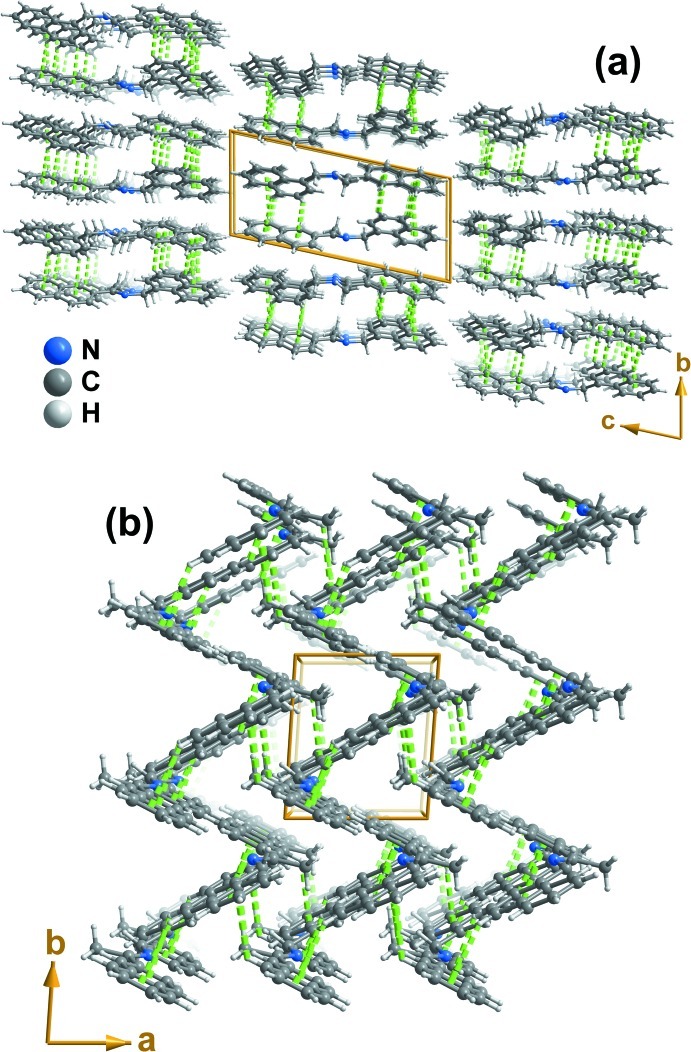
Crystal packing of the title compound viewed in perspective along the (a) [100] and (b) [001] directions of the unit cell. C—H···π supramolecular interactions are depicted as dashed green lines.

## supplementary crystallographic information

### Comment

Imines, azomethines or Schiff bases, are used as synonyms for the same species, with the general form *RR*'C═NR''. These species are generally obtained by condensation of the corresponding primary amines (*R*''NH_2_) with aldehydes (*R*'HC═O) or ketones (*RR*'C═O), and can be additionaly referred to as aldimines and ketimines, respectively (Norton *et al.*, 1954; Hampe *et al.*, 2004). These compounds are stable only when the *R*, *R*', and *R*'' groups are relatively large.

As free ligands, imines have diverse applications: as protecting groups for the C═O double bond or the amine function; as chiral auxiliaries in asymmetric substitution reactions of amino acids; as reagents for the quantitative transformation of aldimines into aza-enolates; the synthesis of primary and secondary amines by reduction of the C═N double bond (Hampe *et al.*, 2004). They can also form complexes with various metals (*e.g.*, Mg, Mn, Co, Cr, Zn Pd, Pt) with application as catalysts of polymerization reactions (*e.g.*, polymerization of lactide; copolymerization of CO_2_ and epoxides), epoxidation of alkenes and for the Heck reaction between methyl acrylate and *p*-iodonitrobenzene (Hampe *et al.*, 2004; Kumar *et al.*, 2008).

The title compound was the isolated product of the reaction of [Eu(nta)_3_(PzPy)] {with Hnta=1-(2-naphthoyl)-3,3,3-trifluoroacetone and PzPy = 2-[3(5)-pyrazolyl]pyridine} with 1-naphthylmethylamine. We believe that this unexpected compound was the product of the reaction of nta^-^ with the amine catalyzed by the presence of the rare-earth metal center. The presence of the metal is essential because the non-catalyzed reaction of β-diketonates with amines does not produce monoimines (Filyakova *et al.*, 1996).

The asymmetric unit of the title compound is composed of a whole molecular unit, C_23_H_19_N (see Scheme and Figure 1). The naphthyl groups are planar with mean e. s. d. from planarity of 0.007 and 0.011 Å, for 1- and 2-naphthyl, respectively. The CH_3_—C═N—CH_2_ moiety is also planar with almost null deviation [mean e.s. d. = 0.002 Å], and the angles subtended by this moiety with the 1-naphthyl and 2-naphthyl groups are 38.69 (11) and 16.50 (11)°, respectively. The close packing of the molecules in the triclinic centrosymmetric space group is mediated by the need to fill the space in conjunction with several supramolecular weak interactions, such as C—H···π (Figure 2; see Table 1 for geometrical details of these supramolecular interactions). Individual molecules are disposed in zigzag-type forming supramolecular layers which are perpendicular to the [100] direction of the unit cell (Figure 2).

### Experimental

All chemicals were purchased from Sigma-Aldrich and used as received. Literature procedures were used to prepare [Eu(nta)_3_(PzPy)] {with Hnta=1-(2-naphthoyl)-3,3,3-trifluoroacetone and PzPy = 2-[3(5)-pyrazolyl]pyridine} (Bruno *et al.*, 2008).

[Eu(nta)_3_(PzPy)] (1.00 g, 0.92 mmol) was dissolved in toluene (45 ml) at ambient temperature. 1-Naphthylmethylamine (0.78 ml, 5.5 mmol) was added leading to the formation of an orange solution, which was refluxed for 3 days. The water formed in the reaction was removed by using a Dean-Stark apparatus. The reaction mixture was filtered off and the solvent removed by evaporation under vacuum, leading to the isolation of an orange oil. Suitable crystals of the title compound were isolated by slow cooling of a concentrated solution in diethyl ether.

Selected FT—IR (KBr, cm^-1^): ν = 3060*m*, 3049*m*, 1630 s, 1620*m*, 1595 s, 1508*m*, 1503s h, 1370*m*, 1357*m*, 1321*m*, 1287*m*, 1263*m*, 1193*m*, 1127*m*, 1080*m*, 1074*m*, 950*m*, 945s h, 897*m*, 864*m*, 829 s, 796vs, 771 s, 746 s, 734 s, 535*m*, 524*m*, 473 s, 405*m*.

^1^H NMR (300 MHz, CDCl_3_, 25 °C): δ = 8.25–7.47 (m, 14H, *H*-naphthyl), 5.24 (s, 2H, CH_2_), 2.53 (s, 3H, CH_3_).

### Refinement

Hydrogen atoms bound to carbon were placed at their idealized positions with C—H = 0.93 Å (aromatic and delocalized), 0.97 Å (—CH_2_—) and 0.96 Å (terminal —CH_3_). These hydrogen atoms were included in the final structural model in riding-motion approximation, with the isotropic thermal displacement parameters fixed at 1.2×*U*_eq_ (for —CH and the —CH_2_— moieties) or 1.5× *U*_eq_ (for the —CH_3_ group) of the carbon atom to which they are attached.

### Crystal data

**Table:**

C_23_H_19_N	*Z* = 2
*M**_r_* = 309.39	*F*(000) = 328
Triclinic, *P*1	*D*_x_ = 1.221 Mg m^−^^3^
Hall symbol: -P 1	Mo *K*α radiation, λ = 0.71073 Å
*a* = 6.6304 (5) Å	Cell parameters from 4208 reflections
*b* = 7.7772 (5) Å	θ = 2.5–24.2°
*c* = 16.7587 (9) Å	µ = 0.07 mm^−^^1^
α = 77.655 (3)°	*T* = 296 K
β = 87.969 (2)°	Block, yellow
γ = 85.734 (3)°	0.17 × 0.07 × 0.04 mm
*V* = 841.69 (9) Å^3^	

### Data collection

**Table:**

Bruker X8 Kappa APEXII CCD diffractometer	2986 independent reflections
Radiation source: fine-focus sealed tube	2021 reflections with *I* > 2σ(*I*)
Graphite monochromator	*R*_int_ = 0.045
ω / φ scans	θ_max_ = 25.2°, θ_min_ = 3.7°
Absorption correction: multi-scan (*SADABS*; Sheldrick, 1998)	*h* = −7→7
*T*_min_ = 0.988, *T*_max_ = 0.997	*k* = −9→9
14750 measured reflections	*l* = −19→20

### Refinement

**Table:**

Refinement on *F*^2^	Primary atom site location: structure-invariant direct methods
Least-squares matrix: full	Secondary atom site location: difference Fourier map
*R*[*F*^2^ > 2σ(*F*^2^)] = 0.044	Hydrogen site location: inferred from neighbouring sites
*wR*(*F*^2^) = 0.121	H-atom parameters constrained
*S* = 1.02	*w* = 1/[σ^2^(*F*_o_^2^) + (0.0562*P*)^2^ + 0.1131*P*] where *P* = (*F*_o_^2^ + 2*F*_c_^2^)/3
2986 reflections	(Δ/σ)_max_ < 0.001
218 parameters	Δρ_max_ = 0.13 e Å^−^^3^
0 restraints	Δρ_min_ = −0.16 e Å^−^^3^

### Special details

**Table:**

Geometry. All e.s.d.'s (except the e.s.d. in the dihedral angle between two l.s. planes) are estimated using the full covariance matrix. The cell e.s.d.'s are taken into account individually in the estimation of e.s.d.'s in distances, angles and torsion angles; correlations between e.s.d.'s in cell parameters are only used when they are defined by crystal symmetry. An approximate (isotropic) treatment of cell e.s.d.'s is used for estimating e.s.d.'s involving l.s. planes.
Refinement. Refinement of *F*^2^ against ALL reflections. The weighted *R*-factor *wR* and goodness of fit *S* are based on *F*^2^, conventional *R*-factors *R* are based on *F*, with *F* set to zero for negative *F*^2^. The threshold expression of *F*^2^ > σ(*F*^2^) is used only for calculating *R*-factors(gt) *etc*. and is not relevant to the choice of reflections for refinement. *R*-factors based on *F*^2^ are statistically about twice as large as those based on *F*, and *R*- factors based on ALL data will be even larger.

### Fractional atomic coordinates and isotropic or equivalent isotropic displacement parameters (Å^2^)

**Table:**

	*x*	*y*	*z*	*U*_iso_*/*U*_eq_	
N1	0.1658 (2)	0.2127 (2)	0.47556 (8)	0.0604 (4)	
C1	−0.1836 (3)	0.2591 (3)	0.52396 (10)	0.0730 (6)	
H1A	−0.2092	0.3849	0.5091	0.109*	
H1B	−0.2531	0.2145	0.5745	0.109*	
H1C	−0.2313	0.2066	0.4819	0.109*	
C2	0.0396 (2)	0.2145 (2)	0.53387 (9)	0.0486 (4)	
C3	0.1216 (2)	0.16403 (19)	0.61818 (9)	0.0452 (4)	
C4	0.3175 (2)	0.0775 (2)	0.63052 (9)	0.0531 (4)	
H4	0.3918	0.0519	0.5859	0.064*	
C5	0.3988 (3)	0.0313 (2)	0.70559 (10)	0.0576 (4)	
H5	0.5274	−0.0258	0.7115	0.069*	
C6	0.2923 (2)	0.0681 (2)	0.77506 (9)	0.0517 (4)	
C7	0.0964 (2)	0.1538 (2)	0.76454 (9)	0.0475 (4)	
C8	0.0156 (2)	0.1991 (2)	0.68525 (9)	0.0485 (4)	
H8	−0.1138	0.2544	0.6784	0.058*	
C9	−0.0116 (3)	0.1929 (2)	0.83342 (10)	0.0598 (5)	
H9	−0.1410	0.2484	0.8273	0.072*	
C10	0.0725 (3)	0.1499 (3)	0.90866 (10)	0.0707 (5)	
H10	0.0004	0.1774	0.9534	0.085*	
C11	0.2652 (3)	0.0652 (3)	0.91923 (11)	0.0745 (6)	
H11	0.3207	0.0363	0.9710	0.089*	
C12	0.3726 (3)	0.0245 (2)	0.85451 (10)	0.0676 (5)	
H12	0.5007	−0.0328	0.8625	0.081*	
C13	0.0991 (3)	0.2558 (3)	0.39124 (9)	0.0681 (5)	
H13A	−0.0210	0.3363	0.3872	0.082*	
H13B	0.0631	0.1490	0.3757	0.082*	
C14	0.2595 (2)	0.33899 (19)	0.33304 (9)	0.0454 (4)	
C15	0.4228 (2)	0.4037 (2)	0.36071 (9)	0.0514 (4)	
H15	0.4369	0.3926	0.4166	0.062*	
C16	0.5702 (3)	0.4866 (2)	0.30687 (11)	0.0628 (5)	
H16	0.6787	0.5318	0.3273	0.075*	
C17	0.5556 (3)	0.5013 (2)	0.22524 (11)	0.0633 (5)	
H17	0.6555	0.5546	0.1902	0.076*	
C18	0.3907 (2)	0.4366 (2)	0.19305 (9)	0.0511 (4)	
C19	0.2387 (2)	0.35522 (19)	0.24721 (9)	0.0454 (4)	
C20	0.0741 (3)	0.2932 (2)	0.21271 (10)	0.0593 (5)	
H20	−0.0269	0.2393	0.2469	0.071*	
C21	0.0605 (3)	0.3109 (3)	0.13055 (10)	0.0720 (5)	
H21	−0.0491	0.2689	0.1094	0.086*	
C22	0.2096 (3)	0.3916 (3)	0.07773 (11)	0.0748 (6)	
H22	0.1989	0.4034	0.0216	0.090*	
C23	0.3697 (3)	0.4527 (3)	0.10818 (10)	0.0678 (5)	
H23	0.4680	0.5065	0.0724	0.081*	

### Atomic displacement parameters (Å^2^)

**Table:**

	*U*^11^	*U*^22^	*U*^33^	*U*^12^	*U*^13^	*U*^23^
N1	0.0528 (9)	0.0836 (11)	0.0417 (8)	−0.0104 (7)	−0.0017 (6)	−0.0044 (7)
C1	0.0554 (11)	0.1075 (16)	0.0545 (11)	0.0058 (10)	−0.0066 (8)	−0.0166 (10)
C2	0.0478 (10)	0.0500 (10)	0.0481 (9)	−0.0084 (7)	−0.0025 (8)	−0.0088 (7)
C3	0.0459 (9)	0.0443 (9)	0.0449 (9)	−0.0070 (7)	−0.0010 (7)	−0.0068 (7)
C4	0.0509 (10)	0.0581 (10)	0.0507 (9)	−0.0014 (8)	0.0007 (7)	−0.0136 (8)
C5	0.0497 (10)	0.0610 (11)	0.0597 (10)	0.0067 (8)	−0.0062 (8)	−0.0100 (8)
C6	0.0569 (10)	0.0467 (10)	0.0496 (9)	−0.0038 (8)	−0.0056 (7)	−0.0055 (7)
C7	0.0531 (10)	0.0432 (9)	0.0451 (9)	−0.0073 (7)	0.0016 (7)	−0.0064 (7)
C8	0.0465 (9)	0.0457 (9)	0.0515 (9)	−0.0023 (7)	−0.0013 (7)	−0.0066 (7)
C9	0.0669 (11)	0.0587 (11)	0.0517 (10)	−0.0020 (9)	0.0057 (8)	−0.0090 (8)
C10	0.0923 (16)	0.0727 (13)	0.0460 (10)	−0.0090 (11)	0.0060 (10)	−0.0103 (9)
C11	0.0926 (15)	0.0816 (14)	0.0464 (10)	−0.0087 (12)	−0.0118 (10)	−0.0041 (9)
C12	0.0712 (12)	0.0705 (12)	0.0564 (11)	0.0004 (10)	−0.0162 (9)	−0.0028 (9)
C13	0.0558 (11)	0.1003 (15)	0.0451 (10)	−0.0165 (10)	−0.0020 (8)	−0.0045 (9)
C14	0.0476 (9)	0.0443 (9)	0.0435 (8)	−0.0005 (7)	−0.0019 (7)	−0.0085 (7)
C15	0.0540 (10)	0.0530 (10)	0.0471 (9)	−0.0033 (8)	−0.0077 (7)	−0.0094 (7)
C16	0.0566 (11)	0.0646 (11)	0.0666 (11)	−0.0172 (9)	−0.0092 (9)	−0.0071 (9)
C17	0.0558 (11)	0.0669 (12)	0.0630 (11)	−0.0149 (9)	0.0056 (8)	−0.0021 (9)
C18	0.0563 (10)	0.0468 (9)	0.0482 (9)	0.0004 (8)	0.0031 (7)	−0.0076 (7)
C19	0.0500 (9)	0.0403 (8)	0.0456 (8)	0.0002 (7)	−0.0024 (7)	−0.0095 (7)
C20	0.0633 (11)	0.0650 (11)	0.0511 (10)	−0.0145 (9)	−0.0032 (8)	−0.0122 (8)
C21	0.0822 (14)	0.0832 (14)	0.0562 (11)	−0.0186 (11)	−0.0112 (10)	−0.0208 (10)
C22	0.0948 (15)	0.0873 (14)	0.0448 (10)	−0.0080 (12)	−0.0032 (10)	−0.0187 (9)
C23	0.0776 (13)	0.0777 (13)	0.0465 (10)	−0.0068 (10)	0.0106 (9)	−0.0107 (9)

### Geometric parameters (Å, º)

**Table:**

N1—C2	1.2650 (19)	C11—H11	0.9300
N1—C13	1.4575 (19)	C12—H12	0.9300
C1—C2	1.502 (2)	C13—C14	1.504 (2)
C1—H1A	0.9600	C13—H13A	0.9700
C1—H1B	0.9600	C13—H13B	0.9700
C1—H1C	0.9600	C14—C15	1.363 (2)
C2—C3	1.494 (2)	C14—C19	1.428 (2)
C3—C8	1.371 (2)	C15—C16	1.403 (2)
C3—C4	1.419 (2)	C15—H15	0.9300
C4—C5	1.352 (2)	C16—C17	1.354 (2)
C4—H4	0.9300	C16—H16	0.9300
C5—C6	1.411 (2)	C17—C18	1.407 (2)
C5—H5	0.9300	C17—H17	0.9300
C6—C7	1.414 (2)	C18—C23	1.412 (2)
C6—C12	1.415 (2)	C18—C19	1.423 (2)
C7—C9	1.413 (2)	C19—C20	1.414 (2)
C7—C8	1.414 (2)	C20—C21	1.360 (2)
C8—H8	0.9300	C20—H20	0.9300
C9—C10	1.362 (2)	C21—C22	1.396 (3)
C9—H9	0.9300	C21—H21	0.9300
C10—C11	1.392 (3)	C22—C23	1.353 (3)
C10—H10	0.9300	C22—H22	0.9300
C11—C12	1.357 (3)	C23—H23	0.9300
			
C2—N1—C13	120.48 (14)	C11—C12—H12	119.5
C2—C1—H1A	109.5	C6—C12—H12	119.5
C2—C1—H1B	109.5	N1—C13—C14	112.15 (13)
H1A—C1—H1B	109.5	N1—C13—H13A	109.2
C2—C1—H1C	109.5	C14—C13—H13A	109.2
H1A—C1—H1C	109.5	N1—C13—H13B	109.2
H1B—C1—H1C	109.5	C14—C13—H13B	109.2
N1—C2—C3	116.57 (14)	H13A—C13—H13B	107.9
N1—C2—C1	124.74 (14)	C15—C14—C19	119.14 (14)
C3—C2—C1	118.67 (14)	C15—C14—C13	121.04 (14)
C8—C3—C4	117.77 (14)	C19—C14—C13	119.80 (14)
C8—C3—C2	122.67 (14)	C14—C15—C16	121.58 (15)
C4—C3—C2	119.55 (14)	C14—C15—H15	119.2
C5—C4—C3	121.60 (15)	C16—C15—H15	119.2
C5—C4—H4	119.2	C17—C16—C15	120.43 (16)
C3—C4—H4	119.2	C17—C16—H16	119.8
C4—C5—C6	121.26 (15)	C15—C16—H16	119.8
C4—C5—H5	119.4	C16—C17—C18	120.57 (16)
C6—C5—H5	119.4	C16—C17—H17	119.7
C5—C6—C7	118.31 (14)	C18—C17—H17	119.7
C5—C6—C12	123.17 (16)	C17—C18—C23	121.84 (16)
C7—C6—C12	118.52 (16)	C17—C18—C19	119.29 (15)
C9—C7—C6	119.01 (15)	C23—C18—C19	118.86 (15)
C9—C7—C8	122.07 (15)	C20—C19—C18	117.78 (14)
C6—C7—C8	118.91 (14)	C20—C19—C14	123.25 (14)
C3—C8—C7	122.14 (15)	C18—C19—C14	118.96 (14)
C3—C8—H8	118.9	C21—C20—C19	121.34 (16)
C7—C8—H8	118.9	C21—C20—H20	119.3
C10—C9—C7	120.45 (17)	C19—C20—H20	119.3
C10—C9—H9	119.8	C20—C21—C22	120.60 (18)
C7—C9—H9	119.8	C20—C21—H21	119.7
C9—C10—C11	120.66 (17)	C22—C21—H21	119.7
C9—C10—H10	119.7	C23—C22—C21	119.98 (17)
C11—C10—H10	119.7	C23—C22—H22	120.0
C12—C11—C10	120.44 (17)	C21—C22—H22	120.0
C12—C11—H11	119.8	C22—C23—C18	121.44 (17)
C10—C11—H11	119.8	C22—C23—H23	119.3
C11—C12—C6	120.91 (18)	C18—C23—H23	119.3
			
C13—N1—C2—C3	178.47 (14)	C7—C6—C12—C11	−0.6 (3)
C13—N1—C2—C1	0.0 (3)	C2—N1—C13—C14	149.71 (16)
N1—C2—C3—C8	164.24 (15)	N1—C13—C14—C15	−15.1 (2)
C1—C2—C3—C8	−17.2 (2)	N1—C13—C14—C19	166.58 (14)
N1—C2—C3—C4	−15.2 (2)	C19—C14—C15—C16	0.3 (2)
C1—C2—C3—C4	163.37 (16)	C13—C14—C15—C16	−177.94 (16)
C8—C3—C4—C5	−0.3 (2)	C14—C15—C16—C17	−1.4 (3)
C2—C3—C4—C5	179.17 (15)	C15—C16—C17—C18	1.2 (3)
C3—C4—C5—C6	−0.3 (3)	C16—C17—C18—C23	178.90 (16)
C4—C5—C6—C7	0.5 (2)	C16—C17—C18—C19	0.0 (3)
C4—C5—C6—C12	−179.04 (16)	C17—C18—C19—C20	179.39 (15)
C5—C6—C7—C9	−179.49 (14)	C23—C18—C19—C20	0.4 (2)
C12—C6—C7—C9	0.1 (2)	C17—C18—C19—C14	−1.0 (2)
C5—C6—C7—C8	0.0 (2)	C23—C18—C19—C14	−179.92 (14)
C12—C6—C7—C8	179.53 (14)	C15—C14—C19—C20	−179.58 (15)
C4—C3—C8—C7	0.8 (2)	C13—C14—C19—C20	−1.3 (2)
C2—C3—C8—C7	−178.68 (14)	C15—C14—C19—C18	0.8 (2)
C9—C7—C8—C3	178.83 (15)	C13—C14—C19—C18	179.11 (15)
C6—C7—C8—C3	−0.6 (2)	C18—C19—C20—C21	−0.2 (2)
C6—C7—C9—C10	0.5 (2)	C14—C19—C20—C21	−179.79 (16)
C8—C7—C9—C10	−178.91 (16)	C19—C20—C21—C22	−0.1 (3)
C7—C9—C10—C11	−0.7 (3)	C20—C21—C22—C23	0.2 (3)
C9—C10—C11—C12	0.2 (3)	C21—C22—C23—C18	0.1 (3)
C10—C11—C12—C6	0.5 (3)	C17—C18—C23—C22	−179.36 (18)
C5—C6—C12—C11	178.97 (17)	C19—C18—C23—C22	−0.4 (3)

### Hydrogen-bond geometry (Å, º)

Cg1, Cg2 and Cg3 are the centroids of the C18–C23, C3–C8 and C6–C12 rings, respectively,

**Table:**

*D*—H···*A*	*D*—H	H···*A*	*D*···*A*	*D*—H···*A*
C9—H9···*Cg*1^i^	0.93	3.00	3.5739 (18)	122
C16—H16···*Cg*2^ii^	0.93	2.84	3.5147 (18)	130
C17—H17···*Cg*3^ii^	0.93	2.84	3.5626 (19)	135

Symmetry codes: (i) −*x*, −*y*+1, −*z*+1; (ii) −*x*+1, −*y*+1, −*z*+1.
